# Population-Based Comparison of Different Risk Stratification Systems Among Prostate Cancer Patients

**DOI:** 10.3389/fonc.2021.646073

**Published:** 2021-04-13

**Authors:** Mu Xie, Xian-Shu Gao, Ming-Wei Ma, Xiao-Bin Gu, Hong-Zhen Li, Feng Lyu, Yun Bai, Jia-Yan Chen, Xue-Ying Ren, Ming-Zhu Liu

**Affiliations:** ^1^ Department of Radiation Oncology, Peking University First Hospital, Beijing, China; ^2^ Department of Radiation Oncology, Zhengzhou University First Affiliated Hospital, Zhengzhou, China

**Keywords:** prostate cancer, comparison, risk stratification system, prostate cancer-specific mortality, population-based

## Abstract

**Background:**

It is not known which risk stratification system has the best discrimination ability for predicting prostate cancer death.

**Methods:**

We identified patients with non-metastatic primary prostate adenocarcinoma diagnosis between 2004 and 2015 using the Surveillance, Epidemiology, and End Results database. Patients were categorized in different risk groups using the three frequently used risk stratification systems of the National Comprehensive Cancer Network guideline (NCCN-g), American Urological Association guideline (AUA-g), and European Association of Urology guideline (EAU-g), respectively. Associations between risk classification and prostate cancer-specific mortality (PCSM) were determined using Kaplan–Meier analyses and multivariable regression with Cox proportional hazards model. Area under the receiver operating characteristics curve (AUC) analyses were used to test the discrimination ability of the three risk grouping systems.

**Results:**

We analyzed 310,062 patients with a median follow-up of 61 months. A total of 36,368 deaths occurred, including 6,033 prostate cancer deaths. For all the three risk stratification systems, the risk groups were significantly associated with PCSM. The AUC of the model relying on NCCN-g, AUA-g, and EAU-g risk stratification systems for PCSM at specifically 8 years were 0.818, 0.793, and 0.689 in the entire population; 0.819, 0.795, and 0.691 in Whites; 0.802, 0.777, and 0.681 in Blacks; 0.862, 0.818, and 0.714 in Asians; 0.845, 0.806, and 0.728 in Chinese patients. Regardless of the age, marital status, socioeconomic status, and treatment modality, AUC of the model relying on NCCN-g and AUA-g for PCSM was greater than that relying on EAU-g; AUC of the model relying on NCCN-g system was greater than that of the AUA-g system.

**Conclusions:**

The NCCN-g and AUA-g risk stratification systems perform better in discriminating PCSM compared to the EAU-g system. The discrimination ability of the NCCN-g system was better than that of the AUA-g system. It is recommended to use NCCN-g to evaluate risk groups for prostate cancer patients and then provide more appropriate corresponding treatment recommendations.

## Introduction

Risk stratification is the cornerstone for clinical decision making for patients with prostate cancer. The D’Amico Risk group Classification (1) classifies patients into low-, intermediate-, and high-risk groups based on pretreatment prostate-specific antigen (PSA) level, biopsy Gleason score (GS), and clinical tumor (T) stage, all variables that are readily available to the treating physician. It was originally developed to estimate the risk of biochemical recurrence (BCR) following treatment for prostate cancer and has become the main standard in clinical practice. A number of key clinical practice guidelines including the National Comprehensive Cancer Network clinical practice guideline (NCCN-g) (2), European Association of Urology guideline (EAU-g) (3), and American Urological Association guideline (AUA-g) (4) are commonly used guidelines for prostate cancer, in which the risk group classifications are based on the D’Amico classification system.

Different classifications exist among the three guidelines. EAU-g is the most similar to the D’Amico stratification system as clinical stage T2c (cT2c) is categorized as high-risk not intermediate-risk, whereas NCCN-g and AUA-g put cT2c in the intermediate-risk group (unless high-risk GS is present or PSA >20 ng/ml). EAU-g does not distinguish between T3–4N0 (no regional lymph node invasion) patients and N1 (regional lymph node involvement) patients within the locally advanced group, whereas these patients are not within the same group in NCCN-g and AUA-g. In EAU-g, intermediate risk patients are not sub-stratified into favorable intermediate and unfavorable intermediate-risk groups as that in NCCN-g and AUA-g. Proportion of positive biopsy cores is considered in classifying favorable and unfavorable intermediate-risk groups according to NCCN-g, but the same thing does not apply to AUA-g. Inconsistent with the NCCN-g recommendations, AUA-g and EAU-g do not distinguish very high-risk patients from high-risk patients. It is unknown which risk classification system performs best in discriminating prostate cancer death. Because risk stratification has important implications for treatment selection and clinical trial enrolment, it is essential to identify the system with the greatest discrimination ability. To our knowledge, only one study (5) exists where a Sweden prostate cancer database was used to compare the prognostic performance of different pretreatment risk stratification tools. The database did not include information about cT2–cT3 substages, and the population was relatively homogeneous. No other studies have compared the prognostic performance of risk classification tools by ethnic group, age, marital status, socioeconomic status, and treatment modality.

To compare the prognostic performance of the NCCN-g, EAU-g, and AUA-g risk stratification systems, we identified prostate cancer patients from a large and racially diverse population-based Surveillance, Epidemiology, and End Results (SEER) database. Prostate cancer specific mortality (PCSM) was used as the primary outcome to test the discrimination ability of the three systems in a large population and specific ethnic groups. We also performed subgroup analysis according to age, marital status, socioeconomic status, and treatment modality to compare the discrimination ability of the three systems.

## Materials and Methods

### Data Source

Data were extracted from the population-based SEER database, including information on patient demographics, primary tumor site, tumor morphology, stage at diagnosis, treatment, and vital status for approximately 27.8% of the U.S. population (6). Data were pulled from 2004 and later as this was the first year complete clinical tumor, node, and metastasis (TNM) stage, GS, and PSA information were available in SEER. All PSA values have undergone quality assurance (7). Complete mortality data are available up to 2015 and therefore the follow-up deadline was December 31, 2015.

### Study Population

A total of 600,692 White, Black, and Asian patients with prostate cancer diagnosis between January 1, 2004 and December 31, 2015 were included. The exclusion criteria were as follows: no positive histology or unknown diagnostic confirmation; prostate was not the first malignant primary site; not adenocarcinoma; diagnosed at death/during autopsy; unknown/unspecific TNM stage, PSA or GS; metastatic; missing/unknown cause of death; unknown follow-up.

### Study Variables

Patients were stratified based on the three different risk grouping methods in NCCN-g 2020 Version 2 (2), EAU-g 2020 (3), and AUA-g 2017 (4), respectively. We classified patients into six risk groups according to the NCCN-g: very low and low-risk (T1–2a and GS ≤ 6 and PSA < 10 ng/ml); favorable intermediate-risk (all of the following: no very-high-risk or high-risk group features; has only one intermediate risk factor (T2b–2c, GS = 7, PSA = 10–20 ng/ml); GS ≤ 6 or = 3 + 4; <50% biopsy cores positive); unfavorable intermediate-risk (no very-high-risk or high-risk group features and at least one of the following: has two or three intermediate-risk factors; GS = 4 + 3; ≥50% biopsy cores positive); high-risk (no very-high-risk features and at least one high-risk feature: T3a or GS = 8–10 or PSA > 20 ng/ml); very high-risk (at least one of the following: T3b–4; Primary Gleason pattern 5; two or three high-risk features); T_any_N1M0 (no distant metastasis). We then classified patients into five risk groups according to the AUA-g: very low- and low-risk (T1–2a and GS ≤ 6 and PSA < 10 ng/ml); favorable intermediate-risk [has no high-risk group features and has at least one intermediate-risk factor (T2b–2c, GS = 7, 10 ≤ PSA < 20 ng/ml); GS ≤ 6 (with 10 ≤ PSA < 20 ng/ml and/or T2b–2c) OR GS = 3 + 4 (with PSA < 10 ng/ml and T1–2)]; unfavorable intermediate-risk (has no high-risk group features and has at least one intermediate-risk factor; GS = 3 + 4 (with either 10 ≤ PSA < 20 ng/ml or T2b–2c) OR GS = 4 + 3]; high-risk (T3–4 or GS = 8–10 or PSA ≥ 20 ng/ml); T_any_N1M0. Finally, we classified patients into four risk groups according to the EAU-g: low-risk (T1–2a and GS ≤ 6 and PSA < 10 ng/ml); intermediate-risk (all of the following: no localized high-risk or locally advanced features; T2b or GS = 7 or PSA 10–20 ng/ml); localized high-risk (no locally advanced features; T2c or GS = 8–10 or PSA > 20 ng/ml); and locally advanced (T3–4N0M0 or T_any_N1M0).

Other variables evaluated include age, race, marital status, Census urban-area based categorization, socioeconomic status (SES), year of diagnosis, and treatment. We divided the treatment modalities into four main categories: radical prostatectomy (RP), radiation therapy (RT), RT but no RP, RT, and RP. There is overlap between the former two groups because some patients had both RP and RT, and the second group consisted of the third and fourth groups. We cannot accurately distinguish between “no radiation therapy” and “unknown if patients received radiation therapy" due to limitations in the treatment data, so we did not identify the patients that had RP but no RT. Patients in our defined “RT” were identified as having had radiation therapy. The specialized Census Tract-level SES and Rurality Database (2000–2015) provided rurality variable and socioeconomic status (SES) index. Census urban-area based categorization, the rurality variable, is the Census Bureau’s percent of the population living in non-urban areas with four categories: All urban (100% urban), mostly urban (≥50% but <100% urban), mostly rural (>0% but <50% urban), and all rural (100% rural tracts). SES index is a time-dependent composite score. It is constructed based on seven variables (8) that measure different aspects of the SES of a census tract (9). They are: Median household income, Median house value, Median rent, Percent below 150% of poverty line, Education Index, Percent working class, and Percent unemployed. The first quintile (group 1) is the 20th centile or less, and the fifth quintile (group 5) corresponds to the 80th centile or higher.

### Statistical Analysis

Mean, standard deviation (SD), median, interquartile range (IQR) or quartile were reported for continuously coded variables. Proportions were calculated for descriptive statistics. The Kaplan–Meier method and the log-rank test were used for survival analyses. Multivariable Cox regression was performed to identify covariates associated with PCSM using age, race, marital status, Census urban-area based categorization, SES, and risk group. The analyses tested the effect of the NCCN-g or EAU-g or AUA-g risk groups on prediction of PCSM. Discrimination ability of NCCN-g, EAU-g, and AUA-g risk stratification systems was tested using the area under the time-dependent receiver operating characteristics (ROC) curve (AUC) (10). Statistical analyses were performed using R version 3.5.2 (R Foundation for Statistical Computing).

## Results

### Patient Characteristics

**Figure 1:**
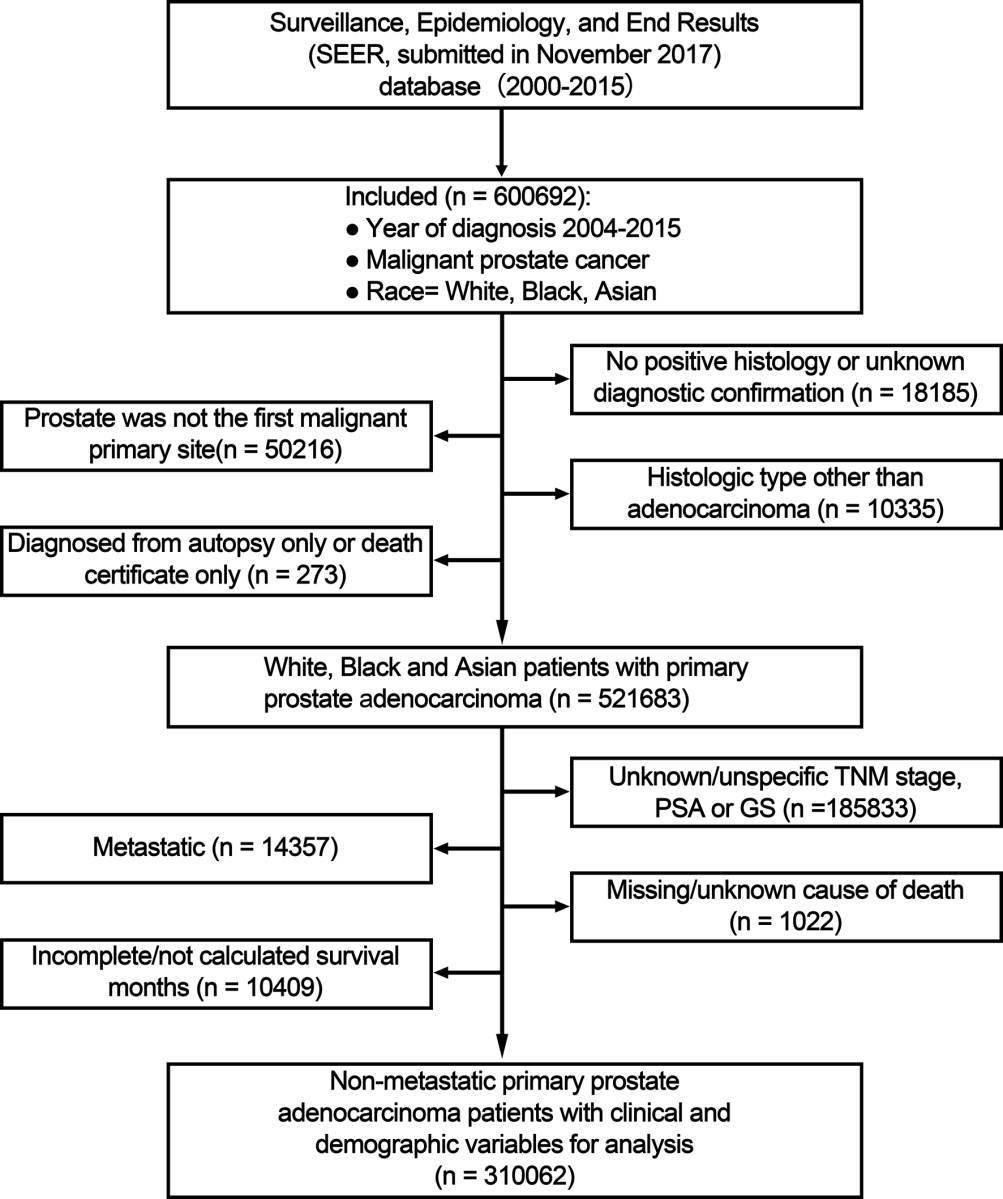
Flow chart for study inclusion and exclusion. PSA, prostate-specific antigen; GS, Gleason score; TNM, tumor, node and metastasis stage.

Baseline patient and tumor characteristics are provided in **Table 1**. A total of 310,062 patients with non-metastatic primary prostate adenocarcinoma diagnosed between 2004 and 2015 were included in the main analysis (**Figure 1**). Mean age was 64.92 (SD: 8.63) years and White patients made up the majority of patients (79.82%), followed by Blacks (15.30%), and Asians (4.88%). 3,073 Chinese made up 20.29% of these Asian patients. Mean PSA level was 9.77 ng/ml. More than 60% of patients had lower PSA levels (<10 ng/ml), GS (≤6 or = 3 + 4) or TNM stage (T1–2bN0M0); thus most patients were categorized into the low and intermediate-risk groups in NCCN-g, or AUA -g, or EAU-g, respectively. 39.69% of patients underwent RP. In the 124,008 patients identified as having received RT, 116,965 patients underwent RT but no RP, and 7,043 patients underwent RT and RP.

**Table 1:** Baseline demographics and clinical characteristics of non-metastatic primary prostate adenocarcinoma, SEER 2004–2015 (Nov 2017 submission).

Patients, N	310,062
Patients died of prostate cancer, n (%)	6,033 (1.95)
Follow-up time, m, median (Quartile)	61 (32, 96)
**Patient demographics**	
Age, y, mean (SD)	64.92 (8.63)
Eligible patients by age, n (%)	
<50	10,437 (3.37)
50**–**54	25,344 (8.17)
55**–**59	48,714 (15.71)
60**–**64	64,536 (20.81)
65**–**69	69,834 (22.52)
70**–**74	48,693(15.70)
75**–**79	27,945 (9.01)
80+	14,559 (4.70)
Race, n (%)	
White	247,486 (79.82)
Black	47,431 (15.30)
Asian	15,145 (4.88)
Marital status, n (%)	
Married	216,151 (69.71)
Others^a^	66,446 (21.43)
unknown	27,465 (8.86)
Census urban-area based categorization, n (%)	
All Rural	18,048 (5.82)
Mostly Rural	20,685 (6.67)
Mostly Urban	63,181 (20.38)
All Urban	207,790 (67.02)
Unknown	358 (0.12)
SES^b^, n (%)	
Group 1	43,075 (13.89)
Group 2	49,757 (16.05)
Group 3	59,048 (19.04)
Group 4	68,862 (22.21)
Group 5	84,119 (27.13)
Unknown	5,201 (1.68)
Year of diagnosis, n (%)	
2004**–**2006	69,269 (22.34)
2007**–**2009	80,915 (26.10)
2010**–**2012	86,036 (27.75)
2013**–**2015	73,842 (23.82)
**Tumor characteristics**	
PSA, ng/ml, mean (SD)	9.77 (12.57)
PSA, n (%)	
<10	238,199 (76.82)
10**–**20	48,441 (15.62)
>20	23,422 (7.55)
GS, n (%)	
≤6	140,619 (45.35)
3 + 4 = 7	88,627 (28.58)
4 + 3 = 7	37,006 (11.94)
8	25,664 (8.28)
9**–**10	18,146 (5.85)
T stage, n (%)	
T1**–**2a	180,219 (58.12)
T2b	8,258 (2.66)
T2c	83,290 (26.86)
T3a	23,125 (7.46)
T3b**–**4	15,170(4.89)
N stage, n (%)	
N0	304,340 (98.15)
N1	5,722 (1.85)
NCCN-g risk group, n (%)	
Very low and low risk	82,530 (26.62)
Favorable Intermediate risk	72,220 (23.29)
Unfavorable Intermediate risk	74,324 (23.97)
High risk	47,758 (15.40)
Very high risk	27,508 (8.87)
T_any_N1M0	5,722 (1.85)
AUA-g risk group, n (%)	
Very low and low risk	82,530 (26.62)
Favorable intermediate risk	81,667 (26.34)
Unfavorable intermediate risk	64,628 (20.84)
High risk	75,515 (24.35)
T_any_N1M0	5,722 (1.85)
EAU-g risk group, n (%)	
Low risk	82,530 (26.62)
Intermediate risk	74,436 (24.01)
Localized high risk	113,149 (36.49)
Locally advanced	39,947 (12.88)
**Treatment**	
Treatment^c^, n (%)	
RP	123,050 (39.69)
RT	124,008 (39.99)
RT but no RP	116,965 (37.72)
RT + RP	7,043 (2.27)

SEER, the Surveillance, Epidemiology, and End Results (SEER) Program provides information on cancer statistics in an effort to reduce the cancer burden among the U.S. population; SD, standard deviations; SES, socioeconomic status; PSA, prostate-specific antigen; GS, Gleason score; T, tumor; N, node; M, metastasis; NCCN-g, National Comprehensive Cancer Network clinical practice guideline; AUA-g, American Urological Association guideline; EAU-g, European Association of Urology guideline; RP, radical prostatectomy; RT, radiation therapy.

a Others include divorced, separated, single (never married), unmarried (or domestic partner), widowed.

b The American National Cancer Institute’s census tract-level socioeconomic status (SES) index is a time-dependent composite score. It is constructed based on seven variables that measure different aspects of the SES of a census tract. They are: Median household income, Median house value, Median rent, Percent below 150% of poverty line, Education Index, Percent working class, and Percent unemployed. After the SES scores are generated for each year, census tracts are categorized into SES quintiles with equal populations in each quintile across the entire SEER catchment area. The first quintile (the group 1) is the 20th centile or less, and the fifth quintile (the group 5) corresponds to the 80th centile or higher.

c There are limitations in the treatment data because we cannot accurately distinguish between “no radiation therapy” and “unknown if patients received radiation therapy”, between curative doses of radiation and palliative radiation therapy, and many factors that determined treatment receipt are not be captured in the registry data. So the number of patients treated with radiation is underestimated. “RP” group includes radical prostatectomy (± radiation therapy), “RT” group includes radiation therapy (± radical prostatectomy).

### Survival Outcomes

Median follow-up was 61 months (IQR: 32–96). A total of 36,368 deaths of any cause occurred, including 6,033 deaths resulting from prostate cancer.

**Figure 2:**
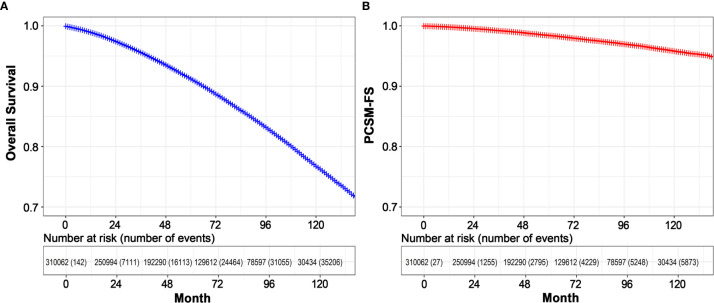
Kaplan**–**Meier estimated overall survival **(A)** and PCSM-FS **(B)** among all patients. PCSM, prostate cancer specific mortality; PCSM-FS, PCSM-free survival.

For all the patients (**Figure 2**), 8-year overall survival (OS) rate was 83.2% (95% CI: 83.0–83.4, P = 0.001), 8-year PCSM-free survival (PCSM-FS) rate was 97.0% (95% CI: 96.9–97.1, P < 0.001).

**Figure 3:**
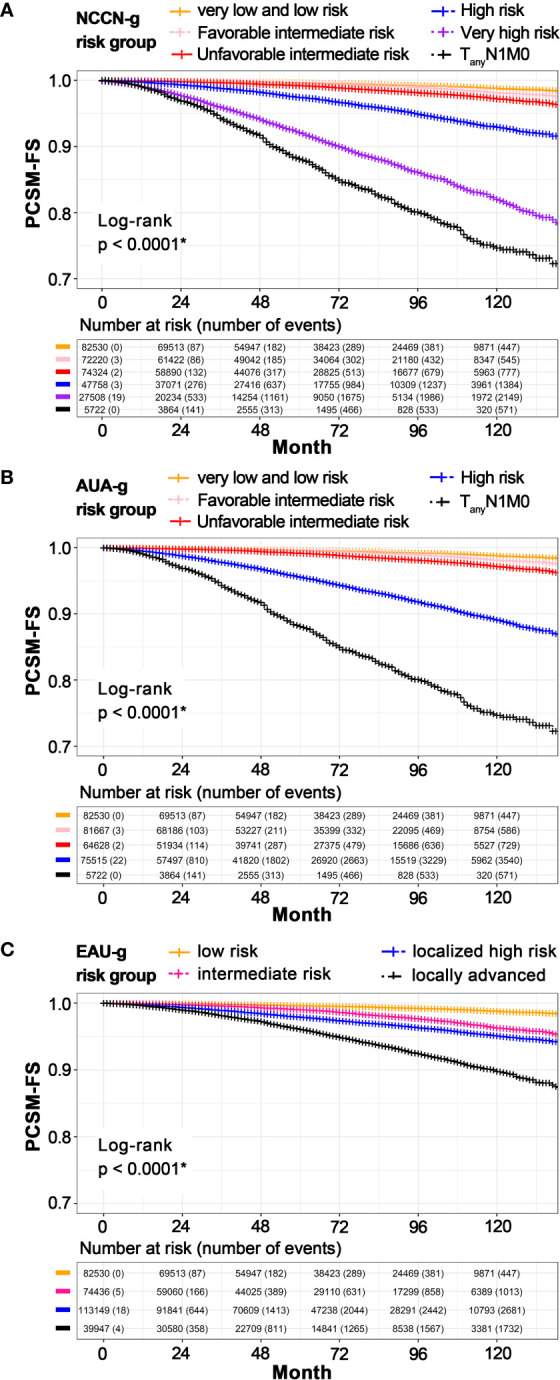
Kaplan**–**Meier estimated PCSM-FS according to risk stratification in NCCN-g **(A)**, AUA-g **(B)**, and EAU-g **(C)**. PCSM, prostate cancer specific mortality; PCSM-FS, PCSM-free survival; NCCN-g, National Comprehensive Cancer Network clinical practice guideline; AUA-g, American Urological Association guideline; EAU-g, European Association of Urology guideline. *Pairwise comparisons using Log-Rank test indicate significant differences between pairs.

Survival estimates as stratified by the three risk-grouping systems are graphically displayed in **Figure 3**. In univariate Kaplan–Meier analyses, 8-year PCSM-FS rates for the six risk groups in NCCN-g were 99.2% (95% CI, 99.1–99.3, P < 0.001), 98.9% (95% CI, 98.8–99.0, P < 0.001), 98.2% (95% CI, 98.0–98.3, P < 0.001), 94.9% (95% CI, 94.6–95.2, P = 0.002), 86.1% (95% CI, 85.5–86.8, P = 0.004), and 80.1% (95% CI, 78.4–81.8, P = 0.011), respectively (**Figure 3A**). In the five AUA-g risk groups, 8-year PCMS-FS rates were 99.2% (95% CI, 99.1–99.3, P < 0.001), 98.9% (95% CI, 98.8–99.0, P < 0.001), 98.1% (95% CI, 98.0–98.3, P < 0.001), 91.8% (95% CI, 91.5–92.1, P = 0.002), and 80.1% (95% CI, 78.4–81.8, P = 0.011), respectively (**Figure 3B**). 8-year PCSM-FS rates for the four risk groups in EAU-g were 99.2% (95% CI, 99.1–99.3, P < 0.001), 97.7% (95% CI, 97.5–97.8, P < 0.001), 96.3% (95% CI, 96.2–96.5, P < 0.001), and 92.5% (95% CI, 92.1–92.9, P = 0.002), respectively (**Figure 3C**).

### Multivariable Regression

On multivariable Cox regression (**Supplementary Table 1**) for PCSM among patients stratified by the NCCN-g risk-grouping system, age 60 to 64 years (*vs* age younger than 50 years; hazard ratio, 1.439; 95% CI, 1.179–1.756; *P* < 0.001), age 65 to 69 years (*vs* age younger than 50 years; hazard ratio, 1.752; 95% CI, 1.438–2.134; *P* < 0.001), age 70 to 74 years (*vs* age younger than 50 years; hazard ratio, 2.467; 95% CI, 2.023–3.007; *P* < 0.001), age 75 to 79 years (*vs* age younger than 50 years; hazard ratio, 4.080; 95% CI, 3.344–4.979; *P* < 0.001), age greater than or equal to 80 years (*vs* age younger than 50 years; hazard ratio, 8.051; 95% CI, 6.603–9.818; *P* < 0.001), Black race (*vs* White; hazard ratio, 1.188; 95% CI, 1.106–1.277; *P* < 0.001), unmarried status (*vs* married status; hazard ratio, 1.426; 95% CI, 1.345–1.512; *P* < 0.001), favorable intermediate-risk (*vs* very low- and low-risk; hazard ratio, 1.410; 95% CI, 1.248–1.594; *P* < 0.001), unfavorable intermediate-risk (*vs* very low- and low-risk; hazard ratio, 2.251; 95% CI, 2.009–2.523; *P* < 0.001), high-risk (*vs* very low- and low-risk; hazard ratio, 5.455; 95% CI, 4.912–6.058; *P* < 0.001), very high-risk (*vs* very low- and low-risk; hazard ratio, 17.262; 95% CI, 15.616–19.081; *P* < 0.001), and T_any_N1M0 (*vs* very low- and low-risk; hazard ratio, 32.168; 95% CI, 28.454–36.366; *P* < 0.001) were significantly associated with an increased PCSM.

Asian race (*vs* White; hazard ratio, 0.700; 95% CI, 0.614–0.798; *P* < 0.001), Census urban-area based categorization “mostly urban” (*vs* “all rural”; hazard ratio, 0.893; 95% CI, 0.798–0.999; *P* = 0.048), group 2 of SES (*vs* group 1; hazard ratio, 0.847; 95% CI, 0.780–0.920; *P* < 0.001), group 3 of SES (*vs* group 1; hazard ratio, 0.741; 95% CI, 0.681–0.806; *P* < 0.001), group 4 of SES (*vs* group 1; hazard ratio, 0.716; 95% CI, 0.659–0.778; *P* < 0.001), and group 5 of SES (*vs* group 1; hazard ratio, 0.590; 95% CI, 0.542–0.642; *P* < 0.001) were significantly associated with a decreased PCSM.

Multivariable Cox regression for PCSM among patients stratified by AUA-g and EAU-g risk-grouping systems are presented in **Supplementary Tables 2**, **3**, and the covariates associated with increased or decreased PCSM are the same as those described above. We summarized the multivariable Cox regression results (for PCSM) of the six risk groups according to NCCN-g, five risk groups according to AUA-g, and four risk groups according to EAU-g in **Table 2**.

**Table 2:** Multivariable cox-regression analyses for PCSM adjusted for age, race, marital status, the Census urban-area based categorization, and SES according to different risk stratification systems.

Covariate	HR	95%CI Lower	95%CI Upper	P
**NCCN-g risk group**				
Very low and low risk	1			
Favorable intermediate risk	1.410	1.248	1.594	0.000
Unfavorable intermediate risk	2.251	2.009	2.523	0.000
High risk	5.455	4.912	6.058	0.000
Very high risk	17.262	15.616	19.081	0.000
T_any_N1M0	32.168	28.454	36.366	0.000
**AUA-g risk group**				
Very low and low risk	1			
Favorable intermediate risk	1.411	1.251	1.591	0.000
Unfavorable intermediate risk	2.304	2.053	2.586	0.000
High risk	9.302	8.445	10.246	0.000
T_any_N1M0	31.714	28.054	35.852	0.000
**EAU-g risk group**				
Low risk	1			
Intermediate risk	2.140	1.919	2.388	0.000
Localized high risk	4.864	4.409	5.367	0.000
Locally advanced	12.444	11.231	13.788	0.000

PCSM, prostate cancer specific mortality; SES, socioeconomic status; HR, hazard ratio; 95%CI, 95% confidence interval; TNM, tumor, node and metastasis; NCCN-g, National Comprehensive Cancer Network clinical practice guideline; AUA-g, American Urological Association guideline; EAU-g, European Association of Urology guideline.

### Comparing Discrimination Ability of the Three Risk Grouping Systems

**Figure 4:**
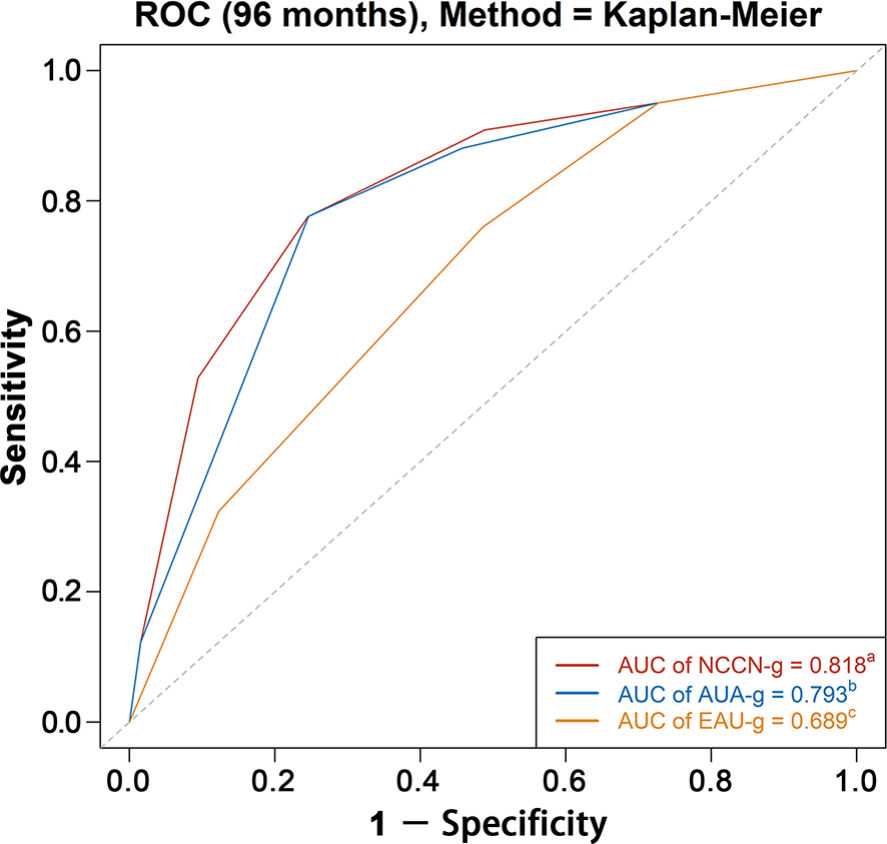
AUC analyses for testing discrimination ability of risk stratification in three guidelines among the entire population. AUC, area under the receiver operating characteristics curve (ROC); NCCN-g, National Comprehensive Cancer Network clinical practice guideline; AUA-g, American Urological Association guideline; EAU-g, European Association of Urology guideline. ^a,b,c^: Different letters indicate significant differences between pairs.

AUC analysis overall and analyses in Whites, Blacks, Asians, and Chinese are graphically displayed in **Figures 4** **,** **5**. The AUC of the model relying on NCCN-g and AUA-g for PCSM at specifically 8 years were 0.818 and 0.793 *versus* 0.689 for EAU-g: 12.9 and 10.4% gain. Similarly, the AUC of the model for PCSM at specifically 8 years in Whites was 0.819 for NCCN-g and 0.795 for AUA-g *versus* 0.691 for EAU-g: 12.8 and 10.4% gain, in Blacks was 0.802 for NCCN-g and 0.777 for AUA-g *versus* 0.681 for EAU-g: 12.1 and 9.6% gain, in Asians was 0.862 for NCCN-g and 0.818 for AUA-g *versus* 0.714 for EAU-g: 14.8 and 10.4% gain, and in Chinese was 0.845 for NCCN-g and 0.806 for AUA-g *versus* 0.728 for EAU-g: 11.7 and 7.8% gain.

**Figure 5:**
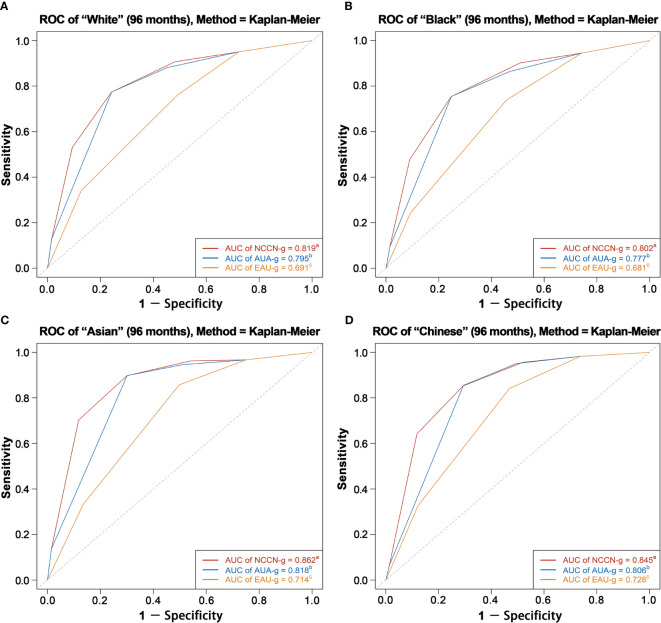
AUC analyses for testing discrimination ability of risk stratification in three guidelines among White patients **(A)**, Black patients **(B)**, Asian patients **(C)**, and Chinese patients **(D)**. AUC, area under the receiver operating characteristics curve (ROC); NCCN-g, National Comprehensive Cancer Network clinical practice guideline; AUA-g, American Urological Association guideline; EAU-g, European Association of Urology guideline. ^a,b,c^: Different letters indicate significant differences between pairs.

In age subgroup analyses (**Supplementary Figure 1**), the discrimination ability of the NCCN-g risk grouping system ranged from 0.787 to 0.907 *vs.* 0.743 to 0.876 for AUA-g system *vs.* 0.710–0.793 for EAU-g system. In marital status subgroup analyses (**Supplementary Figure 2**), the discrimination ability of the NCCN-g risk grouping system ranged from 0.820 to 0.825 *vs.* 0.795 to 0.799 for AUA-g system *vs.* 0.691–0.700 for EAU-g system. In SES subgroup analyses (**Supplementary Figure 3**), the discrimination ability of the NCCN-g risk grouping system ranged from 0.802 to 0.829 *vs.* 0.774 to 0.805 for AUA-g system *vs.* 0.682–0.696 for EAU-g system.

In treatment modality subgroup analyses (**Supplementary Figure 4**), the discrimination ability of the NCCN-g risk grouping system in RP group was 0.920 *vs.* 0.871 for AUA-g system *vs.* 0.821 for EAU-g system; the discrimination ability of the NCCN-g risk grouping system in RT group was 0.848 *vs.* 0.823 for AUA-g system *vs.* 0.801 for EAU-g system; the discrimination ability of the NCCN-g risk grouping system in RT but no RP group was 0.848 *vs.* 0.824 for AUA-g system *vs.* 0.802 for EAU-g system; the discrimination ability of the NCCN-g risk grouping system in RT + RP group was 0.657 *vs.* 0.599 for AUA-g system *vs.* 0.553 for EAU-g system.

## Discussion

In this study, we described the demographics and clinical characteristics of patients with identified non-metastatic primary prostate adenocarcinoma diagnosis and characterized the overall survival and PCSM-free survival among all these patients. We also displayed PCSM-free survival estimates as stratified by risk groups of NCCN-g, AUA-g, and EAU-g systems. We validated that all six risk groups in NCCN-g, five risk groups in AUA-g, and four risk groups in EAU-g are independent prognostic factors of PCSM. We systematically compared the prognostic performance of pretreatment risk stratification systems in the three commonly used guidelines and found that the NCCN-g risk-grouping system had the best discrimination ability; the AUA-g risk-grouping system had the better discrimination ability than the EAU-g system.

Pre-treatment risk stratification of prostate cancer patients enables clinicians to tailor treatment management appropriately and advise patients regarding treatment expectations and probability of disease progression. The risk stratification systems of the three commonly used prostate cancer guidelines were all derived from D’Amico system (1), but they have discordant classification of risk groups. These differences may lead to inappropriate treatment of the same disease condition and make comparison of studies and clinical trial outcomes difficult. We analyzed large and racially diverse patient cohorts in the population-based SEER database to test the discrimination ability of each guideline according clinicopathological information at diagnosis and using the prostate cancer death as the endpoint. In the whole population, our result of comparison is in line with the result of a study conducted in Sweden (5), although information on cT2–cT3 substages was lacking in their database. We also performed subgroup analyses according to race, age, marital status, SES, and treatment modality to determine if the order of performance among the three systems remained the same in subgroups with different characteristics. We found that the NCCN-g and AUA-g risk grouping systems performed better than the EAU-g in predicting PCSM regardless of the race, age, marital status, SES, and treatment modality; the NCCN-g system performed the best.

In EAU-g, cT2c is high-risk, whereas NCCN-g and AUA-g classify cT2c as intermediate-risk (unless high-risk GS is present or PSA is over 20 ng/ml). Differences in classification may determine the extent of lymph node dissection at the time of radical prostatectomy (RP), the duration of androgen deprivation therapy (ADT) given concomitantly with external beam radiation therapy (EBRT), or eligibility for enrolment in clinical trials. Klaassen et al. (11) concluded that patients with cT2c without other high-risk features had similar outcomes to intermediate-risk patients and significantly better outcomes compared to high-risk patients. We analyzed all the 82,579 T2cN0 patients (made up 73.0% patients of localized high-risk group in the EAU-g system) and found that 35.6% of T2cN0 patients were favorable intermediate-risk, and 51.7% were unfavorable intermediate-risk in the NCCN-g system; 41.8% of T2cN0 patients were favorable intermediate-risk, and 45.4% were unfavorable intermediate-risk in the AUA-g system. More importantly, their 5-year, 8-year, and 10-year PCSM-free survival (PCSM-FS) rates (99.3, 98.5, and 97.8%) were even higher than that of intermediate-risk patients (99.0, 97.7, and 96.3%) in EAU-g. These could explain why NCCN-g and AUA-g have better discrimination performance than EAU-g. Categorizing cT2c (without other high-risk features) as high-risk may be unreasonable and could lead to unnecessary extended pelvic lymph node dissection, longer-term ADT (2 to 3 years) in combination with radiation therapy, and inaccurate comparison of clinical outcomes across studies.

Inconsistent with the NCCN-g and AUA-g recommendations, the EAU-g does not distinguish between T3–4N0 patients and N1 patients within the locally advanced group. These patients have markedly distinct prognoses (as supported by our survival analyses results of patients stratified by NCCN-g or AUA-g risk grouping system) and treatment recommendations from one another. This could definitely lower the discrimination ability of the EAU-g stratification to discriminate PCSM.

Intermediate-risk group is heterogeneous with respect to the tumor characteristics and oncological prognoses. Zumsteg et al. (12) separated this group into favorable and unfavorable subsets and found that the unfavorable risk patients who had Grade group 3 disease, or ≥50% positive biopsy cores, or 2–3 intermediate-risk factors had a significant increased risk of biochemical recurrence and PCSM compared with the favorable intermediate-risk patients. NCCN-g stratifies intermediate-risk patients into two subgroups based on the stratification method of the above study, whereas AUA-g subcategorizes the intermediate-risk group into favorable and unfavorable categories of cancer severity based largely on histopathologic GS; the percentage of positive biopsy cores is not considered. Our results demonstrated that the two different stratification methods in NCCN-g and AUA-g could both discriminate PCSM. AUA-g and EAU-g recommend radiotherapy plus ADT for 4–6 months as standard treatment options for patients with intermediate-risk, whereas NCCN-g does not recommend ADT given concomitantly with radiotherapy for favorable intermediate-risk patients. The best stratification and the optimal treatment remain controversial; advanced imaging may further improve current stratification systems of intermediate-risk patients (13). EAU-g does not divide intermediate-risk patients into subgroups, which also explains why its discrimination ability is less than the other two systems.

Differing from AUA-g and EAU-g, NCCN-g subdivided high-risk group into very high- and standard high-risk groups. In general, tools with more detailed risk stratification showed better discrimination. This may explain the better discrimination of the NCCN-g risk grouping system than the AUA-g system. There are some studies defining and validating new classifications of high-risk disease (14–16). For example, Muralidhar et al. (15) found that patients with favorable high-risk prostate cancer (stage T1c with Gleason 4 + 4 = 8 and PSA <10 ng/ml or stage T1c with Gleason 6 and PSA >20 ng/ml) have significantly better PCSM than other patients with high-risk disease and similar PCSM as those with unfavorable intermediate-risk disease, who are typically treated with shorter-course androgen deprivation therapy. New systems may require more detailed classification and personalization of treatment within high-risk disease, which requires more research on associations between risk-subgroup and treatment and prognosis.

Our results showed that comparing with White race, Black race was significantly associated with an increased PCSM, and Asian race was significantly associated with a decreased PCSM. We know that NCCN-g and AUA-g are applicable for the United States and EAU-g for Europe. Whites and Blacks make up the majority of the United States and Europe. So which risk stratification system is the most appropriate for Asians, or even specifically Chinese patients to refer to or use? This is also why we emphasized the racial AUC subgroup analysis over other subgroup analyses. Our answer is the NCCN-g. However, Asian and North American men revealed marked disparities in five-alpha-reductase activity (17, 18), diet intake (19, 20), and mutational landscape of the same disease (21). So even though NCCN-g is of great value to guide risk classification, Asian patients may need a more targeted risk grouping system based on data with Asian characteristics.

In the subgroup analysis of treatment modality, all the three risk grouping systems were weak in discriminating between patients with radical prostatectomy combined radiation therapy (AUCs were all less than 0.7) because more than 80% of the patients with RP combined RT were categorized as high-risk or very high-risk or T1 in NCCN-g, as high-risk or N1 in AUA-g, as localized high-risk or locally advanced in EAU-g.

Our findings suggest that the EAU-g could improve its ability to discriminate PCSM and guide clinical decisions by reclassifying T2c, subdividing the intermediate-risk patients, distinguishing between T3–4N0 and N1, and subdividing the high-risk patients. The need to improve the EAU-g system was prompted by significant differences in prognoses and recommendations pertaining to a breadth of clinical decisions, ranging from advisability of pelvic lymph node dissection during prostatectomy, to advisability of using ADT in conjunction with radiation, to advisability of the duration of ADT given concomitantly with radiation therapy. The best sub-stratification and the corresponding optimal treatments of intermediate-risk group and high-risk group remain controversial; additional credible research is needed.

Current risk stratification methods for prostate cancer, although improved, are far from perfect. Other risk stratification schemas have been proposed and externally validated to provide more accurate risk assessments. The Cambridge Prognostic Groups (CPG) system was developed to predict prostate cancer death accounting for competing events (22, 23). The Cancer of the Prostate Risk Assessment (CAPRA) score provides a predictor of disease recurrence after RP and incorporates not only the standard variables but also the percentage of positive biopsies and patient age into the point-calculated algorithm (24, 25). The Memorial Sloan Kettering Cancer Center (MSKCC) Prostate Cancer nomogram predicts recurrence using a multivariable model (26, 27). A newly developed point-based staging system, the pretreatment clinical prognostic stage group system for non-metastatic prostate cancer by international staging collaboration for cancer of the prostate (STAR-CAP), including T category, N category, primary and secondary GS, pretreatment serum PSA level, percentage of positive core biopsy, and age, has been validated to outperform the NCCN 3-tier, NCCN 4-tier, and CAPRA system in predicting PCSM (28). Although the NCCN-g system performs better than the systems of the other two commonly used guidelines, it does not seem to have a significant advantage over the above risk classification schemas (5, 28). To further improve the risk stratification, additional variables providing independent information and more harmonious incorporation of clinical factors will be needed. Although more recent studies have incorporated genomics and molecular markers to improve prognostication (29–32), they are less generalizable than the staging system including only clinical factors, and the degree to which adding these factors may contribute to improve staging system is unclear.

The main strengths of the current study are the large, racially diverse sample size (~310,000 prostate cancer patients) with detailed clinicopathological data. This study provided a direct comparison among three guidelines, validated the risk groups and presented accuracy testing using the AUC. Our study has several limitations. First, the SEER database does not provide information about the PSA density and the percentage of cancer in each core; thus, we were unable to distinguish very low-risk patients by NCCN-g (2) and AUA-g (4) systems. Therefore, the discrimination abilities of the two systems may be underestimated. Second, information about the percentage of positive biopsy cores among patients diagnosed from 2004 to 2009 is lacking; we estimate that approximately 10,000 to 15,000 unfavorable intermediate-risk patients were incorrectly classified into the favorable intermediate-risk group in the NCCN-g system inevitably, potentially affecting the results of testing the discrimination ability of the NCCN-g system (2). Third, information relating to the cores with Grade Group 4 or 5 is not available in the SEER database; therefore, we likely underestimated the actual number of very high-risk patients in the NCCN-g system (2). Fourth, the pathological data was not centrally reviewed; follow-up was per institutional standards and not prospectively defined. Fifth, census urban-area based categorization and SES were defined at a county level, not an individual level, possibly affecting the results of the Cox regressions. Sixth, there are limitations in the treatment data because we cannot accurately distinguish between “no radiation therapy” and “unknown if patients received radiation therapy”, between curative doses of radiation and palliative radiation therapy; we cannot ascertain if an adequate treatment dose of radiation was given. We cannot get exact information about ADT, chemotherapy, active surveillance, and watchful waiting in SEER, and many factors that determined treatment receipt are not captured in the registry data. Therefore, treatment types was not included in our Cox regression model but was included in subgroup analyses of prognostic performance. Finally, our study relied on cancer registry records; the findings need further validation with independent external cohorts.

## Conclusions

Despite these limitations, our study provides insight into the discrimination abilities of risk stratification systems in the three commonly used guidelines for patients with non-metastatic prostate cancer. It demonstrated the superiority of the NCCN-g and AUA-g systems over the EAU-g system in discriminating PCSM. The discrimination ability of the NCCN-g system was better than that of the AUA-g system. It lends support to using NCCN-g to evaluate risk groups for prostate cancer patients.

## Data Availability Statement

Publicly available datasets were analyzed in this study. This data can be found here: https://seer.cancer.gov/data/.

## Author Contributions

Study conception and design: X-SG and MX. Data acquisition: MX and X-BG. Data and statistical analysis: MX. Drafting of manuscript: MX and M-WM. Critical editorial and writing contributions: H-ZL, FL, YB, J-YC, X-YR, and M-ZL. All authors contributed to the article and approved the submitted version.

## Funding

This work was supported by the Natural Science Foundation of Beijing Municipality (Grant Number: 7182164).

## Conflict of Interest

The authors declare that the research was conducted in the absence of any commercial or financial relationships that could be construed as a potential conflict of interest.

## Supplementary Material

Supplementary Figure 1 AUC analyses for testing discrimination ability of risk stratification in three guidelines among age <50 years patients **(A)**, age 50**–**54 years patients **(B)**, age 55**–**59 years patients **(C)**, age 60**–**64 years patients **(D)**, age 65**–**69 years patients **(E)**, age 70**–**74 years patients **(F)**, age 75**–**79 years patients **(G)**, and age ≥ 80 years patients **(H)**. Abbreviations: AUC, area under the receiver operating characteristics curve (ROC); NCCN-g, National Comprehensive Cancer Network clinical practice guideline; AUA-g, American Urological Association guideline; EAU-g, European Association of Urology guideline. ^a,b,c^: Different letters indicate significant differences between pairs.

Supplementary Figure 2 AUC analyses for testing discrimination ability of risk stratification in three guidelines among married patients **(A)**, and other marital status patients **(B)**. Abbreviations: AUC, area under the receiver operating characteristics curve (ROC); NCCN-g, National Comprehensive Cancer Network clinical practice guideline; AUA-g, American Urological Association guideline; EAU-g, European Association of Urology guideline. ^a,b,c^: Different letters indicate significant differences between pairs.

Supplementary Figure 3 AUC analyses for testing discrimination ability of risk stratification in three guidelines among SES group 1 patients **(A)**, SES group 2 patients **(B)**, SES group 3 patients **(C)**, SES group 4 patients **(D)**, and SES group 5 patients **(E)**. Abbreviations: AUC, area under the receiver operating characteristics curve (ROC); SES, socioeconomic status; NCCN-g, National Comprehensive Cancer Network clinical practice guideline; AUA-g, American Urological Association guideline; EAU-g, European Association of Urology guideline. ^a,b,c^: Different letters indicate significant differences between pairs.

Supplementary Figure 4 AUC analyses for testing discrimination ability of risk stratification in three guidelines among RP patients **(A)**, RT patients **(B)**, RT + RP patients **(C)**, and RT but no RP patients **(D)**. Abbreviations: AUC, area under the receiver operating characteristics curve (ROC); RP, radical prostatectomy; RT, radiation therapy; NCCN-g, National Comprehensive Cancer Network clinical practice guideline; AUA-g, American Urological Association guideline; EAU-g, European Association of Urology guideline. ^a,b,c^: Different letters indicate significant differences between pairs.
