# Supplementary figures and images for: Population-Based Comparison of Different Risk Stratification Systems Among Prostate Cancer Patients
Source: Front Oncol. 2021 Apr 13;11:646073. doi: 10.3389/fonc.2021.646073 (PMC8076565; doi:10.3389/fonc.2021.646073)

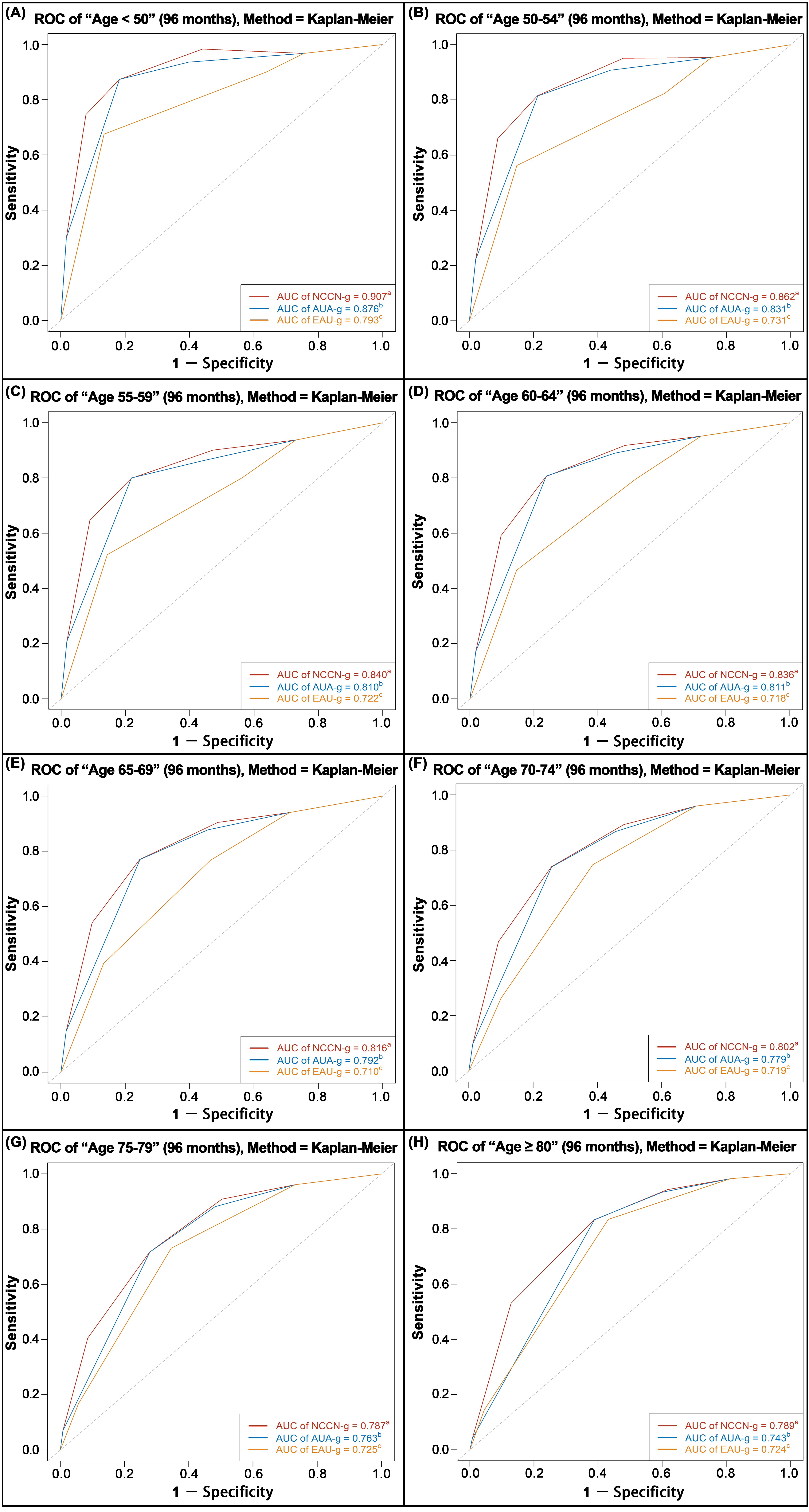

Supplement: Supplementary Figure 1 — AUC analyses for testing discrimination ability of risk stratification in three guidelines among age <50 years patients (A), age 50–54 years patients (B), age 55–59 years patients (C), age 60–64 years patients (D), age 65–69 years patients (E), age 70–74 years patients (F), age 75–79 years patients (G), and age ≥ 80 years patients (H). Abbreviations: AUC, area under the receiver operating characteristics curve (ROC); NCCN-g, National Comprehensive Cancer Network clinical practice guideline; AUA-g, American Urological Association guideline; EAU-g, European Association of Urology guideline. a,b,c: Different letters indicate significant differences between pairs. [file Image_1.tif]

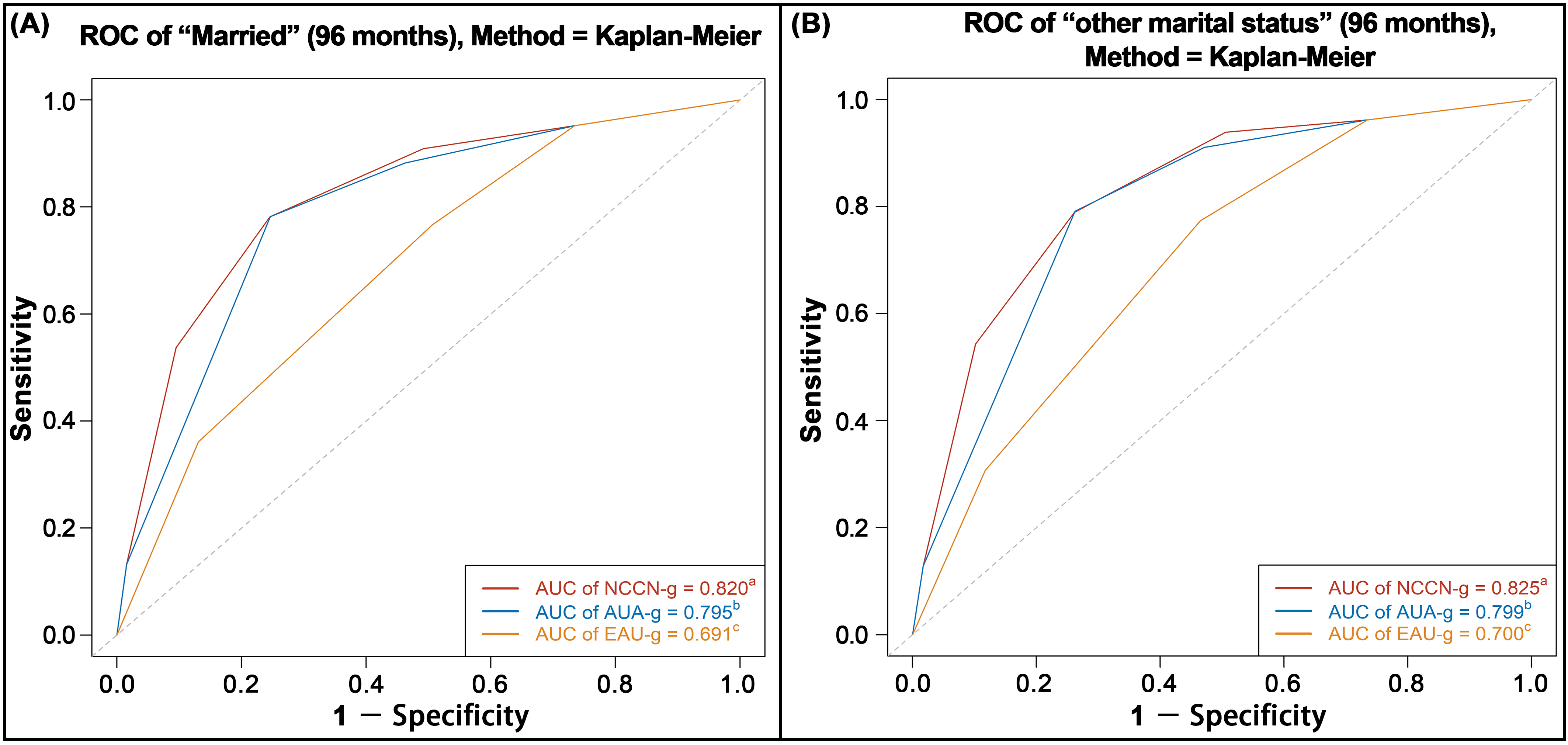

Supplement: Supplementary Figure 2 — AUC analyses for testing discrimination ability of risk stratification in three guidelines among married patients (A), and other marital status patients (B). Abbreviations: AUC, area under the receiver operating characteristics curve (ROC); NCCN-g, National Comprehensive Cancer Network clinical practice guideline; AUA-g, American Urological Association guideline; EAU-g, European Association of Urology guideline. a,b,c: Different letters indicate significant differences between pairs. [file Image_2.tif]

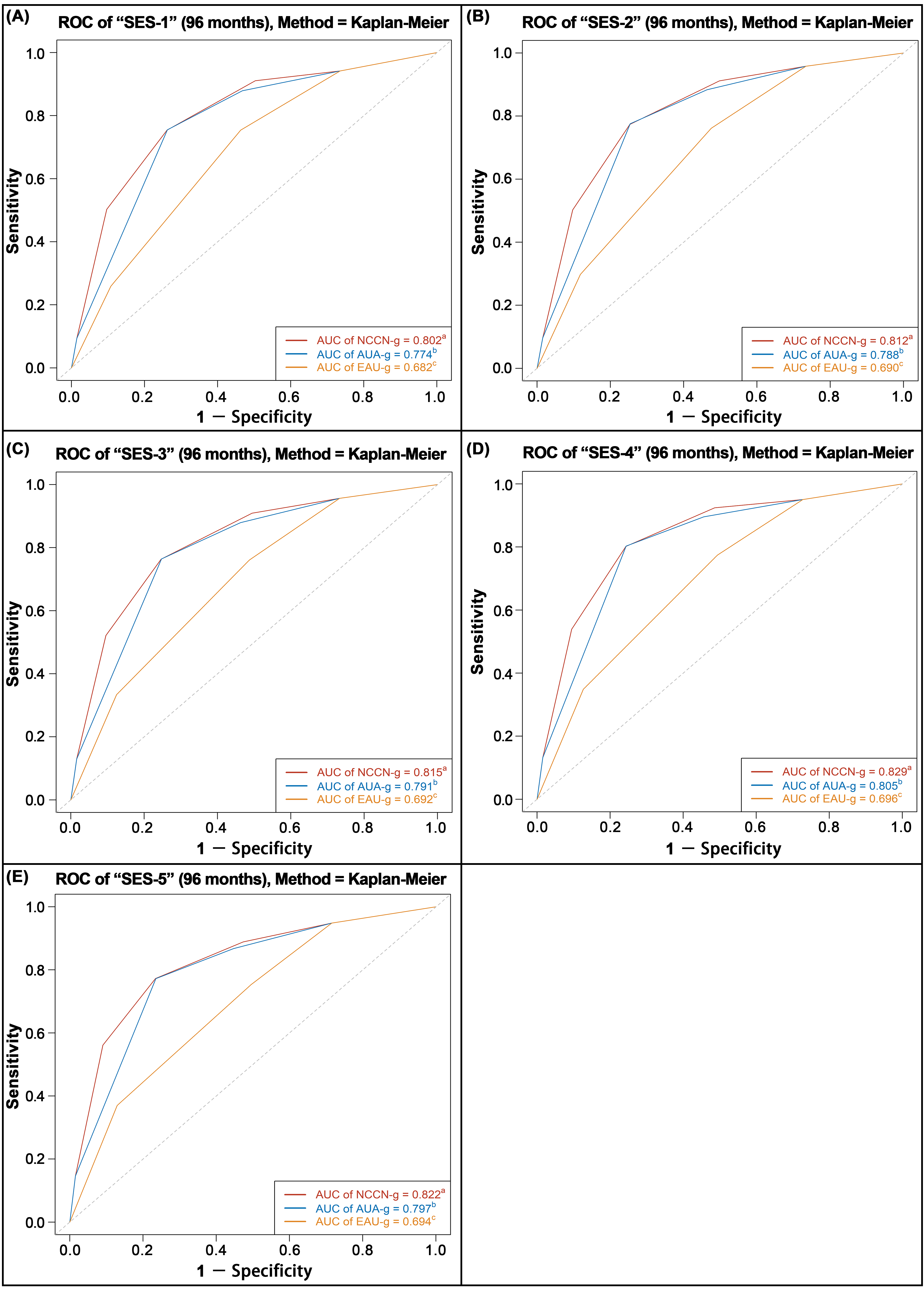

Supplement: Supplementary Figure 3 — AUC analyses for testing discrimination ability of risk stratification in three guidelines among SES group 1 patients (A), SES group 2 patients (B), SES group 3 patients (C), SES group 4 patients (D), and SES group 5 patients (E). Abbreviations: AUC, area under the receiver operating characteristics curve (ROC); SES, socioeconomic status; NCCN-g, National Comprehensive Cancer Network clinical practice guideline; AUA-g, American Urological Association guideline; EAU-g, European Association of Urology guideline. a,b,c: Different letters indicate significant differences between pairs. [file Image_3.tif]

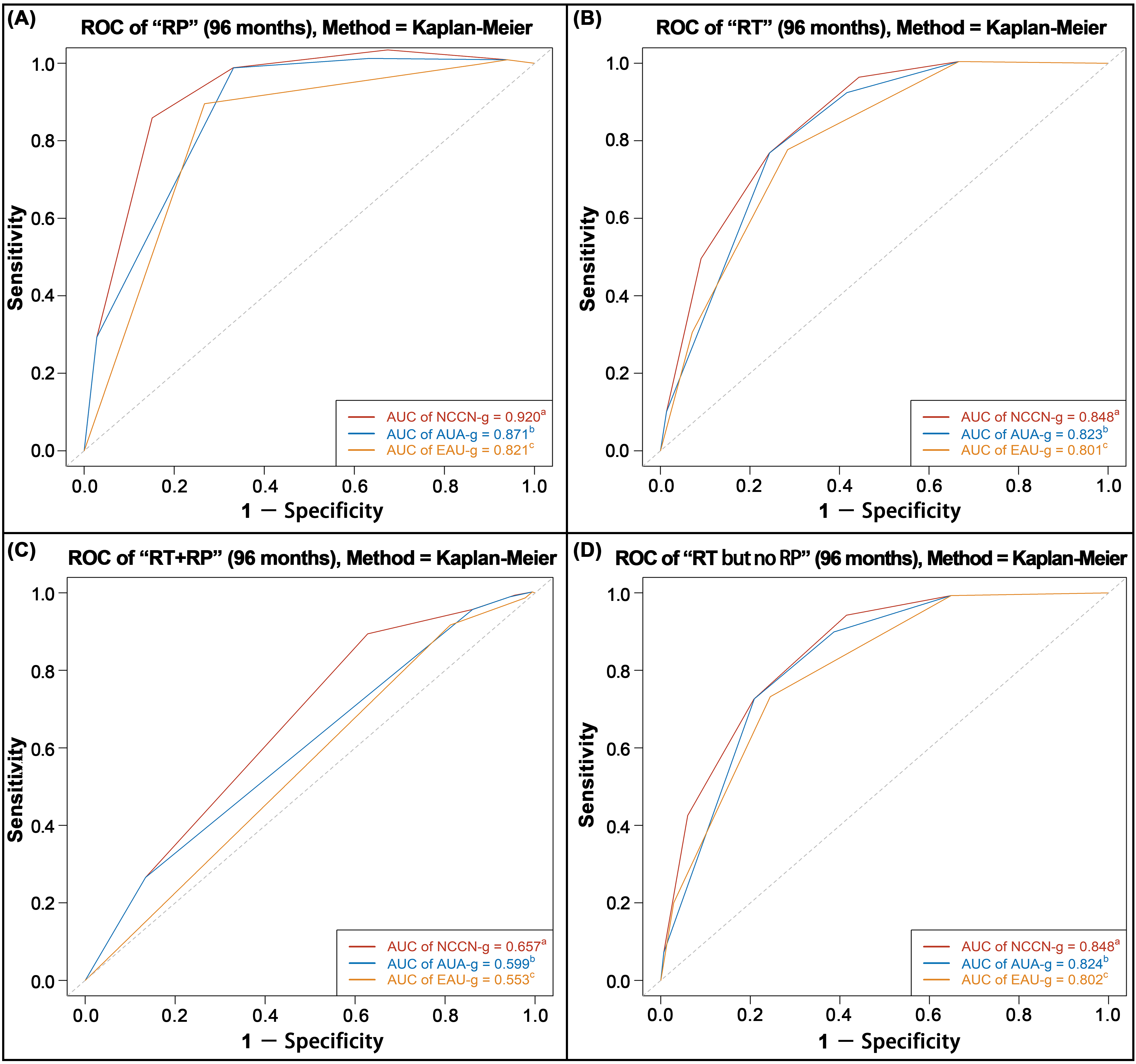

Supplement: Supplementary Figure 4 — AUC analyses for testing discrimination ability of risk stratification in three guidelines among RP patients (A), RT patients (B), RT + RP patients (C), and RT but no RP patients (D). Abbreviations: AUC, area under the receiver operating characteristics curve (ROC); RP, radical prostatectomy; RT, radiation therapy; NCCN-g, National Comprehensive Cancer Network clinical practice guideline; AUA-g, American Urological Association guideline; EAU-g, European Association of Urology guideline. a,b,c: Different letters indicate significant differences between pairs. [file Image_4.tif]
