# Supplementary material for: Population-Based Comparison of Different Risk Stratification Systems Among Prostate Cancer Patients
Source: Front Oncol. 2021 Apr 13;11:646073. doi: 10.3389/fonc.2021.646073 (PMC8076565; doi:10.3389/fonc.2021.646073)
Supplement: Supplementary file 5 [file Table_1.docx]

| **Supplementary table 1. Multivariable cox-regression analyses for PCSM according to NCCN-g risk stratification system** | | | | |
| --- | --- | --- | --- | --- |
| **Covariate** | **HR** | **95%CI Lower** | **95%CI Upper** | **P** |
| Age |  |  |  |  |
| ＜50 | 1 |  |  |  |
| 50-54 | 1.098 | 0.880 | 1.370 | 0.407 |
| 55-59 | 1.195 | 0.974 | 1.467 | 0.088 |
| 60-64 | 1.439 | 1.179 | 1.756 | 0.000 |
| 65-69 | 1.752 | 1.438 | 2.134 | 0.000 |
| 70-74 | 2.467 | 2.023 | 3.007 | 0.000 |
| 75-79 | 4.080 | 3.344 | 4.979 | 0.000 |
| 80+ | 8.051 | 6.603 | 9.818 | 0.000 |
| Race |  |  |  |  |
| White | 1 |  |  |  |
| Black | 1.188 | 1.106 | 1.277 | 0.000 |
| Asian | 0.700 | 0.614 | 0.798 | 0.000 |
| Marital status |  |  |  |  |
| Married | 1 |  |  |  |
| Others ^a^ | 1.426 | 1.345 | 1.512 | 0.000 |
| Census urban-area based categorization |  |  |  |  |
| All Rural | 1 |  |  |  |
| Mostly Rural | 0.900 | 0.786 | 1.031 | 0.127 |
| Mostly Urban | 0.893 | 0.798 | 0.999 | 0.048 |
| All Urban | 0.915 | 0.827 | 1.013 | 0.086 |
| SES ^b^ |  |  |  |  |
| Group 1 | 1 |  |  |  |
| Group 2 | 0.847 | 0.780 | 0.920 | 0.000 |
| Group 3 | 0.741 | 0.681 | 0.806 | 0.000 |
| Group 4 | 0.716 | 0.659 | 0.778 | 0.000 |
| Group 5 | 0.590 | 0.542 | 0.642 | 0.000 |
| **NCCN-g risk group** |  |  |  |  |
| Very low and low risk | 1 |  |  |  |
| Favorable intermediate risk | 1.410 | 1.248 | 1.594 | 0.000 |
| Unfavorable intermediate risk | 2.251 | 2.009 | 2.523 | 0.000 |
| High risk | 5.455 | 4.912 | 6.058 | 0.000 |
| Very high risk | 17.262 | 15.616 | 19.081 | 0.000 |
| T_any_N1M0 | 32.168 | 28.454 | 36.366 | 0.000 |

Abbreviations: PCSM, prostate cancer specific mortality; HR, hazard ratio; 95%CI, 95% confidence interval; SES, socioeconomic status; NCCN-g, National Comprehensive Cancer Network clinical practice guideline.

^a^ Others include divorced, separated, single (never married), unmarried (or domestic partner), widowed.

^b^ The American National Cancer Institute's census tract-level socioeconomic status (SES) index is a time-dependent composite score. It is constructed based on seven variables that measure different aspects of the SES of a census tract. They are: Median household income, Median house value, Median rent, Percent below 150% of poverty line, Education Index, Percent working class, and Percent unemployed. After the SES scores are generated for each year, census tracts are categorized into SES quintiles with equal populations in each quintile across the entire SEER catchment area. The first quintile (the group 1) is the 20th centile or less, and the fifth quintile (the group 5) corresponds to the 80th centile or higher.

| **Supplementary table 2. Multivariable cox-regression analyses for PCSM according to AUA-g risk stratification system** | | | | |
| --- | --- | --- | --- | --- |
| **Covariate** | **HR** | **95%CI Lower** | **95%CI Upper** | **P** |
| Age |  |  |  |  |
| ＜50 | 1 |  |  |  |
| 50-54 | 1.078 | 0.864 | 1.345 | 0.508 |
| 55-59 | 1.168 | 0.951 | 1.433 | 0.139 |
| 60-64 | 1.403 | 1.149 | 1.712 | 0.001 |
| 65-69 | 1.699 | 1.394 | 2.070 | 0.000 |
| 70-74 | 2.338 | 1.918 | 2.850 | 0.000 |
| 75-79 | 3.742 | 3.067 | 4.566 | 0.000 |
| 80+ | 7.597 | 6.230 | 9.264 | 0.000 |
| Race |  |  |  |  |
| White | 1 |  |  |  |
| Black | 1.174 | 1.092 | 1.261 | 0.000 |
| Asian | 0.713 | 0.626 | 0.813 | 0.000 |
| Marital status |  |  |  |  |
| Married | 1 |  |  |  |
| Others ^a^ | 1.431 | 1.349 | 1.517 | 0.000 |
| Census urban-area based categorization |  |  |  |  |
| All Rural | 1 |  |  |  |
| Mostly Rural | 0.888 | 0.776 | 1.018 | 0.087 |
| Mostly Urban | 0.864 | 0.772 | 0.966 | 0.011 |
| All Urban | 0.890 | 0.805 | 0.985 | 0.025 |
| SES ^b^ |  |  |  |  |
| Group 1 | 1 |  |  |  |
| Group 2 | 0.853 | 0.786 | 0.927 | 0.000 |
| Group 3 | 0.747 | 0.687 | 0.813 | 0.000 |
| Group 4 | 0.724 | 0.666 | 0.787 | 0.000 |
| Group 5 | 0.593 | 0.545 | 0.646 | 0.000 |
| **AUA-g risk group** |  |  |  |  |
| Very low and low risk | 1 |  |  |  |
| Favorable intermediate risk | 1.411 | 1.251 | 1.591 | 0.000 |
| Unfavorable intermediate risk | 2.304 | 2.053 | 2.586 | 0.000 |
| High risk | 9.302 | 8.445 | 10.246 | 0.000 |
| T_any_N1M0 | 31.714 | 28.054 | 35.852 | 0.000 |

Abbreviations: PCSM, prostate cancer specific mortality; HR, hazard ratio; 95%CI, 95% confidence interval; SES, socioeconomic status; AUA-g, [American Urological Association](http://www.baidu.com/link?url=QdoNNlwasaponFgae2LfLBRiyDnVGC3KAhO6NUFUnRC) guideline.

^a^ Others include divorced, separated, single (never married), unmarried (or domestic partner), widowed.

^b^ The American National Cancer Institute's census tract-level socioeconomic status (SES) index is a time-dependent composite score. It is constructed based on seven variables that measure different aspects of the SES of a census tract. They are: Median household income, Median house value, Median rent, Percent below 150% of poverty line, Education Index, Percent working class, and Percent unemployed. After the SES scores are generated for each year, census tracts are categorized into SES quintiles with equal populations in each quintile across the entire SEER catchment area. The first quintile (the group 1) is the 20th centile or less, and the fifth quintile (the group 5) corresponds to the 80th centile or higher.

| **Supplementary table 3. Multivariable cox-regression analyses for PCSM according to EAU-g risk stratification system** | | | | |
| --- | --- | --- | --- | --- |
| **Covariate** | **HR** | **95%CI Lower** | **95%CI Upper** | **P** |
| Age |  |  |  |  |
| ＜50 | 1 |  |  |  |
| 50-54 | 1.179 | 0.945 | 1.471 | 0.145 |
| 55-59 | 1.331 | 1.084 | 1.634 | 0.006 |
| 60-64 | 1.700 | 1.392 | 2.075 | 0.000 |
| 65-69 | 2.206 | 1.810 | 2.688 | 0.000 |
| 70-74 | 3.428 | 2.811 | 4.179 | 0.000 |
| 75-79 | 6.231 | 5.104 | 7.607 | 0.000 |
| 80+ | 14.524 | 11.907 | 17.716 | 0.000 |
| Race |  |  |  |  |
| White | 1 |  |  |  |
| Black | 1.303 | 1.212 | 1.400 | 0.000 |
| Asian | 0.757 | 0.664 | 0.863 | 0.000 |
| Marital status |  |  |  |  |
| Married | 1 |  |  |  |
| Others ^a^ | 1.528 | 1.441 | 1.620 | 0.000 |
| Census urban-area based categorization |  |  |  |  |
| All Rural | 1 |  |  |  |
| Mostly Rural | 0.893 | 0.780 | 1.023 | 0.102 |
| Mostly Urban | 0.880 | 0.786 | 0.984 | 0.025 |
| All Urban | 0.890 | 0.804 | 0.985 | 0.024 |
| SES ^b^ |  |  |  |  |
| Group 1 | 1 |  |  |  |
| Group 2 | 0.844 | 0.777 | 0.916 | 0.000 |
| Group 3 | 0.721 | 0.663 | 0.784 | 0.000 |
| Group 4 | 0.698 | 0.642 | 0.758 | 0.000 |
| Group 5 | 0.560 | 0.514 | 0.609 | 0.000 |
| **EAU-g risk group** |  |  |  |  |
| low risk | 1 |  |  |  |
| Intermediate risk | 2.140 | 1.919 | 2.388 | 0.000 |
| Localized high risk | 4.864 | 4.409 | 5.367 | 0.000 |
| Locally advanced | 12.444 | 11.231 | 13.788 | 0.000 |

Abbreviations: PCSM, prostate cancer specific mortality; HR, hazard ratio; 95%CI, 95% confidence interval; SES, socioeconomic status; EAU-g, European Association of Urology guideline.

^a^ Others include divorced, separated, single (never married), unmarried (or domestic partner), widowed.

^b^ The American National Cancer Institute's census tract-level socioeconomic status (SES) index is a time-dependent composite score. It is constructed based on seven variables that measure different aspects of the SES of a census tract. They are: Median household income, Median house value, Median rent, Percent below 150% of poverty line, Education Index, Percent working class, and Percent unemployed. After the SES scores are generated for each year, census tracts are categorized into SES quintiles with equal populations in each quintile across the entire SEER catchment area. The first quintile (the group 1) is the 20th centile or less, and the fifth quintile (the group 5) corresponds to the 80th centile or higher.
